# The Walnuts and Healthy Aging Study (WAHA): Protocol for a Nutritional Intervention Trial with Walnuts on Brain Aging

**DOI:** 10.3389/fnagi.2016.00333

**Published:** 2017-01-10

**Authors:** Sujatha Rajaram, Cinta Valls-Pedret, Montserrat Cofán, Joan Sabaté, Mercè Serra-Mir, Ana M. Pérez-Heras, Adam Arechiga, Ricardo P. Casaroli-Marano, Socorro Alforja, Aleix Sala-Vila, Mónica Doménech, Irene Roth, Tania M. Freitas-Simoes, Carlos Calvo, Anna López-Illamola, Ella Haddad, Edward Bitok, Natalie Kazzi, Lynnley Huey, Joseph Fan, Emilio Ros

**Affiliations:** ^1^Center for Nutrition, Healthy Lifestyle and Disease Prevention, School of Public Health, Loma Linda University, Loma LindaCA, USA; ^2^Lipid Clinic, Endocrinology and Nutrition Service, Hospital Clínic, Institut d’Investigacions Biomèdiques August Pi i SunyerBarcelona, Spain; ^3^Centro de Investigación Biomédica en Red Fisiopatología de la Obesidad y Nutrición, Instituto de Salud Carlos IIIMadrid, Spain; ^4^Department of Psychology, School of Behavioral Health, Loma Linda University, Loma LindaCA, USA; ^5^Ophthalmology Service, Hospital Clínic, Institut d’Investigacions Biomèdiques August Pi i SunyerBarcelona, Spain; ^6^Department of Ophthalmology, School of Medicine, Loma Linda University, Loma LindaCA, USA

**Keywords:** cognitive decline, Alzheimer’s disease, age-related macular degeneration, dietary intervention, walnuts, randomized trial, aging

## Abstract

**Introduction:** An unwanted consequence of population aging is the growing number of elderly at risk of neurodegenerative disorders, including dementia and macular degeneration. As nutritional and behavioral changes can delay disease progression, we designed the Walnuts and Healthy Aging (WAHA) study, a two-center, randomized, 2-year clinical trial conducted in free-living, cognitively healthy elderly men and women. Our interest in exploring the role of walnuts in maintaining cognitive and retinal health is based on extensive evidence supporting their cardio-protective and vascular health effects, which are linked to bioactive components, such as n-3 fatty acids and polyphenols.

**Methods:** The primary aim of WAHA is to examine the effects of ingesting walnuts daily for 2 years on cognitive function and retinal health, assessed with a battery of neuropsychological tests and optical coherence tomography, respectively. All participants followed their habitual diet, adding walnuts at 15% of energy (≈30–60 g/day) (walnut group) or abstaining from walnuts (control group). Secondary outcomes include changes in adiposity, blood pressure, and serum and urinary biomarkers in all participants and brain magnetic resonance imaging in a subset.

**Results:** From May 2012 to May 2014, 708 participants (mean age 69 years, 68% women) were randomized. The study ended in May 2016 with a 90% retention rate.

**Discussion:** The results of WAHA might provide high-level evidence of the benefit of regular walnut consumption in delaying the onset of age-related cognitive impairment and retinal pathology. The findings should translate into public health policy and sound recommendations to the general population (ClinicalTrials.gov identifier NCT01634841).

## Introduction

The worldwide expansion of aging populations has become a major public health challenge (Song and Chen, 2015). Increased lifespan has resulted in a steady rise of debilitating diseases related to aging, including neurodegenerative disorders, such as Alzheimer’s disease (AD), the most common type of dementia (Alzheimer’s Association, 2015), and age-related macular degeneration (AMD), the leading cause of visual loss and blindness worldwide (Lim et al., 2012). Unfortunately, to date there is no effective treatment for AD (Iqbal et al., 2014) or even mild cognitive impairment (MCI), its precursor stage (Cooper et al., 2013). Likewise, no effective preventive strategies exist for AMD, although nutritional and behavioral modifications can delay disease progression once initiated (Lim et al., 2012). In the continuum from normal cognition to MCI and dementia there is age-related cognitive decline, the onset and progression of which depends on a number of factors, including genetic variation, the level of education, environmental factors, particularly dietary habits, and the presence of cardiovascular risk factors or disease (Baumgart et al., 2015). Similar risk factors, albeit with a different genetic background, play a role in the pathophysiology of AMD (Lim et al., 2012). Optimal cognitive and visual functions are essential for quality of life and developing effective primary prevention strategies to reduce the economic and societal burden of such common age-related diseases would be of utmost public health importance.

Oxidative stress and inflammation are thought to play a pivotal role in precipitating neurodegenerative diseases, including AD (Schrag et al., 2013; Heneka et al., 2015) and AMD (Khandhadia and Lotery, 2010). Consequently, as derived from epidemiologic studies, a promising hypothesis in recent years has been that antioxidant-rich foods, such as fruit, vegetables, and particularly seeds and berries (Nooyens et al., 2011; Valls-Pedret et al., 2012; Ros and Hu, 2013; Barbour et al., 2014; O’Brien et al., 2014), and plant-based dietary patterns, especially the Mediterranean diet and the DASH (Dietary Approaches to Stop Hypertension) dietary pattern (Otaegui-Arrazola et al., 2014; Tangney et al., 2014) may protect from cognitive decline and AD. Nutrients such as n-3 polyunsaturated fatty acids (n-3 PUFA) and antioxidants, namely polyphenols, carotenoids and vitamins C and E, may have a role in preventing both cognitive impairment (Macready et al., 2009; Dangour et al., 2012; Valls-Pedret et al., 2012; Devore et al., 2013; Barnes et al., 2014; Otaegui-Arrazola et al., 2014) and macular degeneration (Age-Related Eye Disease Study Research Group, 2001; Parisi et al., 2008; Tan et al., 2009; Majumdar and Srirangam, 2010; Merle et al., 2013). Nuts in general and walnuts in particular have a rich matrix of these bioactive components and through additive effects have the potential to beneficially impact neuronal function in the brain and the retina (Poulose et al., 2014). Prospective studies have provided support for the association between nut consumption and improved cognitive performance (Valls-Pedret et al., 2012; O’Brien et al., 2014) and lower incidence of AMD (Tan et al., 2009; Amirul Islam et al., 2014). Among the different tree nuts, walnuts contain n-3 PUFA, specifically α-linolenic acid (ALA; C18:3n-3). ALA has long been believed to improve brain health indirectly via modest conversion to docosahexaenoic acid (DHA; C22:6n-3) (Domenichiello et al., 2015), which has been found to modulate brain plasticity and counteract neuroinflammation in experimental studies (Dyall, 2015). However, studies in rodents uncovered brain benefits of ALA by itself, including increased brain plasticity (Blondeau et al., 2009), reduced cell death and calcium dysregulation (Carey et al., 2013), and reduced amyloid-beta deposition (Gao et al., 2016). Other bioactive compounds in walnuts, such as arginine, tocopherols, folate, melatonin, and polyphenols also support neurological health and cognitive wellness by modulating blood pressure, HDL function, glucoregulation, endothelial vasodilator function, arterial compliance, oxidative status, and vascular inflammation (Carey et al., 2013; Del Rio et al., 2013; O’Brien et al., 2014). In support, experimental feeding studies in aged rodents have shown that walnuts improve age-related motor and cognitive deficits as assessed by rod walk, plank walk, and Morris water maze tests (Willis et al., 2009).

Easy to implement lifestyle modifications that might help prevent chronic non-communicable disorders need to be uncovered, with the concept that even small changes in the right direction can lead to substantial benefit for global health. Thus, delaying the onset of AD by only 5 years would significantly reduce its prevalence by the next decade (OECD, 2014). Thus far randomized controlled trials (RCTs) testing various interventions in patients with established AD have failed to show any benefit, probably because at this stage neuropathology is far advanced and irreversible. This underlines the need to conduct RCTs in individuals at risk but cognitively healthy, when there are little or no underlying brain changes.

Although not primarily focused on neurodegenerative disorders, one such study, the PREvención con DIeta MEDiterránea (PREDIMED) study, is a landmark RCT conducted in older individuals at high cardiovascular risk that has shown beneficial effects of Mediterranean diets supplemented with extra-virgin olive oil or mixed nuts on several age-related disorders after intervention for ∼5 years (Martínez-González et al., 2015). In the PREDIMED trial, the Mediterranean diets reduced the incidence of cardiovascular diseases by 30%, compared with a low-fat control diet, while the risk of stroke was reduced by 34% by the olive oil diet and by 49% by the nuts diet (Estruch et al., 2013). In a PREDIMED sub-study, we showed better cognitive performance associated with consumption of polyphenol-rich Mediterranean foods at baseline (Valls-Pedret et al., 2012) and cognitive improvement in participants allocated the two Mediterranean diets compared with those in the control group after 4.1 years of follow-up (Valls-Pedret et al., 2015). Thus, data from PREDIMED suggest that nuts, including walnuts, are a good option for cardiovascular and brain health. Therefore, it is reasonable to surmise that usual walnut consumption may be an effective approach to preserve cognitive abilities and visual function. However, while there is sufficient rationale for the role of walnuts in neuroprotection, direct clinical evidence is lacking. The Walnuts and Healthy Aging (WAHA) study is the first RCT assessing cognitive function and macular health in an elderly cohort following daily ingestion of walnuts for 2 years.

## Methods

### Study Design

The WAHA study is a dual center, single blind, randomized 2-year clinical trial conducted in free living, cognitively healthy elderly individuals^1^. The study is carried out in two centers: Loma Linda University, CA, USA (LLU) and Hospital Clínic, Barcelona, Spain (BCN). With a parallel design, participants were randomized to either the walnut group (consuming walnuts daily) or control group (abstaining from walnuts). Otherwise participants followed their habitual diet throughout the study. Primary aims are to assess changes in cognitive function and retinal integrity. Secondary aims relate to effects on cardio-metabolic risk factors, body weight and composition, and circulating markers of oxidation/inflammation. Additional secondary aims (BCN center) are changes in brain magnetic resonance imaging (MRI), ultrasound-assessed carotid atherosclerosis, blood pressure by 24-h ambulatory monitoring, bone mineral density, leukocyte telomere length, and microRNAs (miRNAs) related to lipoprotein metabolism.

### Participants and Eligibility Criteria

Recruitment and selection of participants took place between May 2012 and May 2014; the trial ended May 31, 2016. Participants were healthy elderly men and women with normal cognitive and visual function at the time of recruitment. Inclusion criteria were age between 63 and 79 years, apparently healthy, and equally willing to be in either of the two groups. Exclusion criteria included inability to undergo neuropsychological testing; morbid obesity (BMI ≥ 40 kg/m^2^); uncontrolled diabetes (HbA1c > 8%); uncontrolled hypertension (on-treatment blood pressure ≥ 150/100 mmHg); prior stroke, significant head trauma or brain surgery; relevant psychiatric illness; major depression; cognitive deterioration or dementia with a score < 24 on the Mini-Mental State Examination (MMSE) (Folstein et al., 1975); other neurodegenerative disorders like Parkinson’s disease; advanced AMD or eye-related conditions precluding ophthalmological evaluation; prior chemotherapy; chronic illness with projected shortened lifespan; allergy to walnuts; customary use of fish oil and/or tree nuts (> 2 servings/week) and/or other relevant sources of ALA, such as flaxseed oil or soy lecithin.

Eligible participants were recruited via mailing study brochures (LLU) or through the non-profit organization Institute of Aging (BCN), advertisements in the study centers, and word of mouth. Interested individuals attended an informational group meeting, completed a short medical questionnaire and signed the informed consent. Next candidates had a face-to-face interview with the study clinician, who assessed potential compliance, reviewed the medical history, inclusion and exclusion criteria, and recent blood work and use of medications or supplements, and administered the MMSE. Eligible participants were scheduled to have baseline tests (neuropsychological and ophthalmologic evaluations and collection of fasting blood and urine) and were then randomized to either the control or walnut group using a computerized random number table with stratification by center, sex, and age range. Couples entering the study were treated as one number and were randomized into the same group.

### Intervention

Participants received 15% of daily energy intake as walnuts for 2 years or abstained from walnuts. To estimate the required amount of walnuts, participants completed a 3-day diet history and a physical activity questionnaire at baseline. The physical activity factor and the energy requirements were obtained using the World Health Organization formula for energy needs for adults > 60 years (World Health Organization, 1985). The estimated amount of walnuts ranged from 1 to 2 oz/day (≈30–60 g/day). Sachets for daily consumption containing 30, 45, or 60 g of raw, pieced walnuts were provided as 8-week allotments to the participants in the walnut group at the time of their 2-monthly clinic visits with the dietitians. Instructions were given to eat walnuts daily, preferably as the raw product, either as a snack or by incorporating them into shakes, yogurts, cereals, or salads. To improve participants’ compliance, 1-kg extra walnut allowances were provided every 2 months to take into account family needs. Participants in the control group were advised to abstain from eating walnuts for the duration of the study. Use of ALA-rich canola and soybean oils was restricted.

Once randomized, participants were scheduled for 2-month visits with the study dietitians aimed at assessing compliance, increasing retention, and collecting data on diet adherence, medication changes, anthropometry, and clinical blood pressure. For the walnut group participants, the dietitians noted any side effects and collected used walnut sachets as a measure of compliance. The rapport built by the dietitians with the participant during the periodic scheduled visits was critical to retain them for the entire length of study. Since participants in the control group had no active intervention, to improve retention, quarterly educational group activities unrelated to the study were offered to them.

### Outcomes

Primary outcomes are changes from baseline in the composite score of all neuropsychological tests for cognition and in the average thickness of the retinal nerve fiber layer of each eye, as assessed by optical coherence tomography (OCT). As secondary cognitive outcomes, we will analyze composites for different cognitive domains, including memory, language, perception, and frontal functions (for further detail, please see section Cognitive Testing); assess the incidence of AD according to NINCDS-ADRDA criteria (McKhann et al., 1984); and evaluate the incidence of MCI, defined by Petersen’s diagnostic criteria (Petersen et al., 2001). Secondary OCT outcomes are macula cube thickness, cube volume, and central thickness.

Other secondary outcomes for the whole cohort are changes in anthropometric measures, lipid profile, serum inflammatory and oxidative stress markers, red blood cell (RBC) fatty acids, and urinary polyphenols. At the BCN site, the following secondary outcomes will be assessed: structural and functional brain MRI variables, carotid intima-media thickness (IMT) and plaque burden, body composition, bone mineral density, 24-h ambulatory blood pressure, leukocyte telomere length, and serum miRNAs.

### Measurements

**Table 1** shows the variables measured in the WAHA trial and how often they were measured.

**Table 1:** Measurements in the Walnuts and Healthy Aging (WAHA) study.

Measurements	Content	Baseline	Year 1	Year 2
Eligibility questionnaire	Sex, age, inclusion, and exclusion criteria	X		
General/quality of life questionnaire^∗^	Marital and socioeconomic status, medical conditions, medication	X		X
Follow-up questionnaire, including withdrawal	Symptoms and conditions, tolerance, medication changes		X	X
Food frequency questionnaire (FFQ)^†^	Multiple food groups and foods	X		X
Diet recalls^‡^	All daily foods and beverages	X	X	X
Physical activity questionnaire	Short-version of the Minnesota questionnaire	X	X	X
Clinical blood pressure		X	X	X
Electrocardiogram^¶^		X		X
Anthropometric measurements	Height, body weight, waist, and hip circumferences, % body fat^§^	X	X	X
Neuropsychological evaluation	A complete test battery to assess cognitive function	X		X
Ophthalmological examination	Optic nerve and macular optical coherence tomography	X		X
Standard blood chemistry	Lipid profile, glucose, insulin, renal function, liver function, blood count, others	X	X	X
Serum inflammatory and oxidative stress markers	CRP, E-selectin, others	X		X
Urine chemistry	Albumin, total polyphenols, ellagitannin, urolithins, others	X	X	X
Red blood cell fatty acids	Measure of compliance, performed in 30% of the cohort	X	X	X
Genomic studies	*APOE*, others	X		
Brain MRI^¶^	Performed in a subset (*n* = 120)	X		X
B-mode ultrasonography of carotid arteries^¶^	Carotid intima-media thickness and plaque burden	X		X
DEXA^§^	Body composition and bone mineral density	X		X
Twenty-four-hour ambulatory blood pressure monitoring^¶^	Performed in 80% of the cohort	X		X
Leukocyte telomere length^¶^	Performed in all participants at baseline and only in those with brain MRI at 1 and 2 years	X	X	X
Serum miRNAs^¶^	miRNAs involved in lipoprotein metabolism	X	X	

*BCN, Barcelona; LLU, Loma Linda University; CRP, C-reactive protein; MRI, magnetic resonance imaging; DEXA, dual-energy X-ray absorptiometry; miRNAs, microRNAs.*

*^∗^The general questionnaire was collected at baseline at both sites. At LLU this questionnaire was called the Quality of Life questionnaire and was collected at end of year 2 also and included more questions about the subjects’ mental health and well-being.*

*^†^Forty-four-food groups and items FFQ at baseline and year 2 at BCN; 150-food group and items FFQ once during the 2nd year at LLU.*

*^‡^Three-day (including one weekend day) self-recorded recalls in BCN at baseline, years 1 and 2; five unannounced 24-h recalls by telephone at LLU over 2 years.*

*^§^ Percent body fat measured by bioelectric impedance at LLU and by DEXA in BCN.*

*^¶^ Determined only at the BCN center.*

#### Measurements at Both Sites

##### Dietary assessment

In BCN, a food frequency questionnaire (FFQ) including 44 food groups was administered to all participants at baseline and end of year 2. In LLU, after the 1st study year, participants filled in a FFQ validated in a pilot sample of elderly persons (Segovia-Siapco et al., 2007). During the study, dietary intake was monitored by conducting five unannounced 24-h diet recalls over 2 years (LLU) or 3-day food records every 6 months (BCN). Participants allocated to the walnut diet who had difficulty chewing due to dental problems were given a coffee grinder at no cost, with instructions on how to consume the ground walnuts by incorporating them to semifluid foods such as yogurt.

##### Clinical evaluation

A clinician saw the participants at baseline, when a general questionnaire was filled in, and at the end of the study, when a follow-up questionnaire was administered to assess final changes in clinical status and medication and adverse events. In the same visit clinical blood pressure was measured following the recommendations of the European Societies of Hypertension and Cardiology (ESH/ESC) (Mancia et al., 2013): after a 5-min rest with the patient seated in a quiet environment, three measures of BP were taken at 2-min intervals with a validated semiautomatic oscillometer (Omron 705-CP, Omron Healthcare Group, Kyoto, Japan). The mean of the last two measurements of systolic and diastolic blood pressure was recorded as office blood pressure. An electrocardiogram (ECG) was performed at baseline and end of study.

##### Physical activity, anthropometry, and body composition

Physical activity was evaluated at baseline, year 1, and the end of the study with a validated short version of the Minnesota questionnaire (Elosua et al., 1994). The dietitians measured height at baseline and 2 years using a wall mounted stadiometer, body weight at baseline and every 2 months by calibrated scales, and waist and hip circumferences every 6 months by using an anthropometric tape midway between the lowest rib and at the iliac crest and at the widest point in the hips, respectively. Body composition was assessed at baseline and 2 years by bioelectric impedance using a body composition analyzer (Tanita, Model TBF-300A, Arlington Heights, IL, USA) at LLU and by dual energy X-ray absorptiometry (DEXA) (Whole body scanner GE-Lunar IDXA, GE Healthcare, Madison, WI, USA) at BCN.

##### Cognitive testing

A comprehensive neuropsychological test battery evaluating several cognitive domains was administered at baseline and at the end of the trial. Neuropsychologists who were masked to participant’s group assignment conducted the cognitive tests. The instruments used were Block design from the Wechsler Adult Intelligence Scale (WAIS III) (Wechsler, 1997), Rey-Osterrieth Complex Figure (ROCF) (Rey, 1941), Rey Auditory Verbal Learning Test (RAVLT) (Rey, 1958), Boston Naming Test (Kaplan et al., 2001), Semantic category evocation of animals (Ramier and Hécaen, 1970), Number location and incomplete letters from the Visual Object and Space Perception Battery (VOSP) (Warrington and James, 1991), Trail Making Test, parts A and B (Partington and Leiter, 1949), Phonemic fluency (FAS) (Benton and Hamsher, 1976), Stroop Color Word Test (Stroop, 1935), Symbol Digit Modalities Test (SDMT) (Smith, 1973), Digit span forward and backward from the WAIS III (Wechsler, 1997), and the Conners Continuous Performance Test (CPT II) (Conners and Staff, 2000).

Subjects’ raw test punctuations were standardized to *z* scores to generate a global cognition composite by computing the mean standardized changes of all neuropsychological tests. This composite was pre-specified as the primary outcome of the study. Moreover, composites of cognitive domains analyzed separately were calculated. First, the memory composite included the mean standardized individual change scores of the RAVLT (immediate and delayed recall) and the 3-min recall of ROCF. Language composite included scores from animal semantic fluency and the Boston Naming Test. Perception composite included scores from number location and incomplete letters from VOSP battery and block design from WAIS-III battery. Finally, a composite score related to frontal functions was created including scores from TMT parts A and B, FAS, Stroop, SDMT, digit span from WAIS-III, and CPT-II. As secondary outcomes, composites assessing cognitive domains separately were defined.

In addition, information about cognitive reserve and mood was collected using a cognitive reserve questionnaire (Solé-Padullés et al., 2009) and the Hamilton Depression Rating Scale (Hamilton, 1967), respectively. Premorbid intelligence was assessed with the American National Adult Reading Test (Grober and Sliwinski, 1991) at LLU site and the Word Accentuation Test (Del Ser et al., 1997) at BCN site.

##### Ophthalmologic evaluation

Retinal examinations were performed at baseline and end of the study by ophthalmologists (BCN) and trained technicians under the supervision of an ophthalmologist (LLU). The central and average thickness and volume of the macular neurosensorial retina and the thickness of the retinal nerve fiber layer were ascertained by OCT (Cirrus HD OCT, Zeiss, Germany).

##### Biochemical measurements

Fasting blood samples and morning spot urine samples were collected at baseline and end of years 1 and 2, and aliquots of EDTA plasma, serum, buffy coat for DNA recovery, whole blood, and urine samples are kept frozen at -80°C. Participants reported on the assigned days after fasting for a minimum of 12 h. An experienced phlebotomist drew blood and samples were centrifuged and aliquoted for the various assays and stored immediately at -80°C. All assays except routine chemistry for safety assessment and determination of the *APOE* genotype were performed at the end of the study to control for between-assay variability. Extra aliquots were stored for additional outcomes of interest to the investigators that might arise during or after the completion of the study. Samples were shipped overnight on dry ice to the appropriate laboratories for biochemical determinations once the study was completed. Plasma and/or serum samples will be used to determine changes in lipid profile, glycemic control, liver and renal function tests, and inflammation and oxidation biomarkers. Extra aliquots of all samples have been stored at -80°C. To reduce assay variability, all samples for a specific assay will be run together in the same laboratory.

An objective biological marker of compliance with walnut consumption, the RBC fatty acid proportions of ALA (Sala-Vila et al., 2011), was assessed in a randomly selected subset (30% of participants) at baseline and years 1 and 2. Given that walnuts have one of the highest polyphenol content of all edible plants (Carey et al., 2013), the urinary content of total polyphenols (Valls-Pedret et al., 2012) will also be used as an indirect marker of compliance.

##### Genetic testing

The *APOE* genotype will be determined by using the method of Hixson and Vernier (Hixson and Vernier, 1990).

#### Measurements Only at BCN Site

Some techniques were performed only at the BCN center based on prior expertise, availability, and cost.

##### Brain magnetic resonance imaging

Approximately 120 participants underwent brain MRI at baseline and 2 years using a 3-tesla scanner (Magnetom Trio Tim, Siemens, Germany). The protocol included high-resolution 3D structural datasets, a diffusion tensor imaging sequence, a Pulsed Arterial Spin Labeling (PASL)-MRI perfusion sequence, a fluid attenuated inversion recovery (FLAIR) image sequence, and a functional sequence with an n-back working memory task.

##### Carotid ultrasonography

Bilateral carotid artery ultrasound imaging was performed at baseline and 2 years according to a standardized protocol (Sala-Vila et al., 2014). Main outcome measurements were mean and maximum carotid IMT and plaque presence, maximum height and area. Briefly, patients underwent sonographic assessment with an Acuson X300 ultrasound system (Siemens, Germany) equipped with a VF 10-5 linear multifrequency transducer (frequency range 5–10 MHz) and ECG synchronization. The same certified sonographer performed all examinations without knowledge of group allocation. Secondary outcomes were mean and maximum IMT at each carotid segment. IMT was defined as the average of multiple distance readings between the far wall lumen–intima and media-adventicia interfaces taken bilaterally at common carotid artery 1 cm pre-bifurcation, bifurcation, and internal carotid artery 1 cm after the flow divider. Plaques were sought by using B-mode and color Doppler examinations in both longitudinal and transverse planes to take into consideration circumferential asymmetry and were defined as a focal wall thickening encroaching into the arterial lumen by at least 50% of the surrounding IMT value or with thickness of at least 1.5 mm as measured from the media adventitia interference to the intima-lumen surface. IMT and plaque measurements were taken oﬄine by using edge-finding software in the predefined segments of the arterial wall. Plaque height was recorded at the more appropriate view, either longitudinal or transversal. Plaque area was determined by the technique of Spence et al. (2002). Plaque burden was recorded for all study subjects and defined in two ways: the sum of maximum heights of all plaques and the sum of areas of all plaques.

The same certified sonographer blinded to treatment allocation performed all scan readings. Consistency (reliability or repeatability) of ultrasound carotid wall measurements was evaluated by comparing results from repeated examinations in 14 subjects performed 3 days apart. Intraclass correlation coefficient ranged from 0.92 to 0.96 for IMT mean (average of right and left) and IMT maximum (maximum value from either right or left) in common, bulb, and internal carotid segments.

##### DEXA studies

Total body lean and fat mass distribution and bone mineral density were assessed by DEXA at baseline and 2 years. This is a standard procedure performed using the whole-body scanner GE Lunar iDXA (GE Healthcare, Madison, WI, USA) according to the manufacturer’s specifications. The iDXA is a narrow fan-beam DXA instrument with a high weight limit (204 kg) and a relatively wide scanning space (66 cm) designed to accommodate obese subjects. The subjects were positioned in the center of the table for each scan. The appropriateness of patient’s position was further assessed by the DXA software by means of an automatic detection system. The instrument has three scan modes that adjust the X-ray attenuation for the thickness of each patient. For this study, scans were performed using the default scan mode automatically selected by the DXA software. The GE Lunar Body Composition Software was used to obtain fat-free mass (FFM) and fat mass (FM) measurements, as well as segmental FM and FFM distribution (trunk, right and left upper limb, right and left lower limb, android, and gynoid) (Hull et al., 2009).

##### Ambulatory blood pressure monitoring

Twenty-four-hour ambulatory blood pressure was monitored in 67% of participants selected at random at baseline and 2 years, following the ESH/ESC recommendations (Mancia et al., 2013; Doménech et al., 2014). The devices were Spacelabs 90207/90217 (Spacelabs^®^ Inc., Richmond, WA, USA), programmed for blood pressure lectures every 20 min during the day and every 30 min at night. Each participant kept a diary of daily activities and times of going to bed and waking up. The report included the duration of monitoring (h), the proportion of valid blood pressure registers, and the mean values of systolic and diastolic blood pressure during periods of activity, rest and the total recording period.

##### Telomere length

Leukocyte telomere length was determined in all participants at baseline and in those undergoing sequential brain MRI at the end of years 1 and 2. Fresh peripheral blood mononuclear cells were separated from 5 mL of EDTA-collected blood by Ficoll gradient centrifugation and also stored at -80°C until determination of telomere length by HTQFISH, which involves *in situ* hybridization of telomere repeats to fluorescent primer and analysis by fluorescence quantification of confocal images.

##### MicroRNA

Finally, at baseline and 1 year circulating miRNAs potentially involved in lipoprotein metabolism will be screened by qRT-PCR in plasma samples. The procedure requires a sample of ∼1 mL plasma per measurement, kept in Trizol^®^ and stored at -80°C until the moment of the analysis.

### Statistical Analyses

Sample size calculation was based on prior results from the PREDIMED study (Valls-Pedret et al., 2015). We used as reference the 2-year unadjusted changes in RAVLT (total learning) scores in the Mediterranean diet enriched with nuts compared to the control diet. In order to have a 90% power to detect differences in the contrast with the null hypothesis, assuming mean changes of 1.05 and 2.10 points in the control and intervention groups, respectively, with a standard deviation of 4.00, the total number of participants required was 308 per group. Considering an estimated dropout rate of 10%, we needed to include a total of 686 participants.

All data collected in the study were entered into an online database (Onto CRF, Costaisa, Spain) managed for both centers. An oversight committee blinded to subject allocation monitored safety, quality of data collected, and trial’s progress.

Between-group differences in baseline neuropsychological and ophthalmologic data will be examined by ANOVA and further by ANCOVA adjusting for center, sex, age, education years, *APOE4* genotype, smoking, BMI, energy intake, physical activity, diabetes, hyperlipidemia, and hypertension. Changes for each individual cognitive test, cognitive composites, ophthalmologic evaluations, and foods and nutrients ensuing intervention will be assessed by ANOVA and further by ANCOVA adjusting for the variables listed above. Statistical significance will be set at the *P* < 0.05 level. Analyses will be performed using SPSS software, version 16.0 (IBM Corp., New York, NY, USA).

## Stepwise Procedures

**FIGURE 1:**
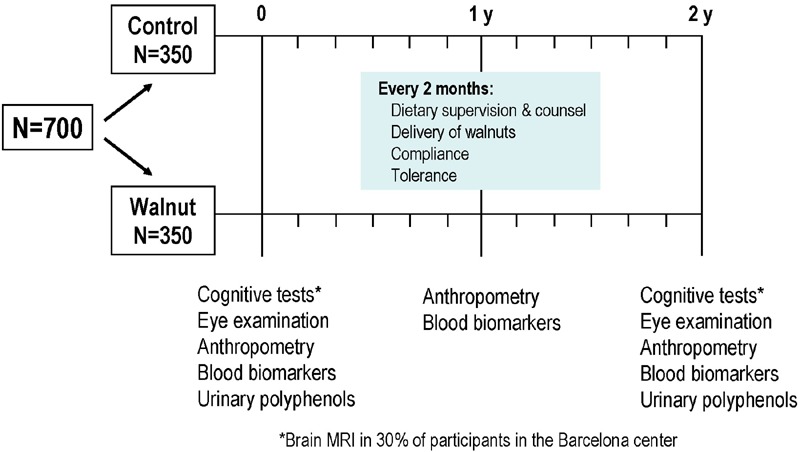
**Design of the Walnuts and Healthy Aging (WAHA) study**.

As depicted in **Figure 1**, there are two main points of data collection, at baseline and at the end of the study after 2 years of intervention. However, there are intermediate points wherein relevant information is obtained. Thus, in mid-study (12 months) a physical activity questionnaire was administered and blood was collected for determination of safety biochemistry analyses, lipid profile, and some secondary outcome variables such as leukocyte telomere length. Nutritional information and office blood pressure was obtained every 6 months, and general health status, tolerance and side effects, medication changes, and anthropometric data were ascertained every 2 months.

The pre-intervention visit with the study’s clinician lasted 1 h and consisted of a face-to-face interview with the candidate wherein complete information on the trial was provided, medical information was collected, inclusion/exclusion criteria were recorded, office blood pressure was measured, an ECG was performed, and the MMSE were administered. If the candidate was suitable for the trial and willing to participate, the informed consent was signed and blood extraction and urine collection and the first neuropsychologist and ophthalmologic visits were scheduled for next week. In the Barcelona site, one part of the informed consent concerned brain MRI, and candidates were asked to fill it in if willing to undergo the procedure. Once the pre-established number of 30% of the cohort was reached, no further candidates were offered brain MRI.

The neuropsychologist visit included the comprehensive test battery described in the Section “Cognitive Testing,” which took 90 min on average to complete. Whenever a cognitive alteration was detected at this visit, the candidate was excluded from further studies and was referred to the local Neurology clinic with a report describing the test findings. In the Barcelona site, the neuropsychologist provided full details on the brain MRI examination to willing candidates and scheduled the procedure.

In the ophthalmologic visit, which lasted approximately 30 min, a general history of eye health was taken and the OCT was performed. Any candidate found to have advanced AMD or bilateral cataracts precluding retinal examination was excluded from the study and referred to the local Ophthalmology clinic.

Once candidates were considered fit to enter the study after assessment of the main outcomes, they were scheduled for the first visit with the dietitian wherein they were randomized to the walnut or control diets. Participants allocated to the walnut diet received their first 2-month allotment of walnuts plus extra packs for family needs. In the BCN site, after the first dietitian visit all participants were scheduled for the secondary outcome variables carotid ultrasound, DEXA for both body composition and bone mineral density, and ambulatory blood pressure monitoring. During the 2 years of the study, dietitians took care of all aspects of follow-up at the bimonthly visits. These included dietary compliance, tolerance and side-effects, anthropometric measurements, delivery of walnuts and recount of empty packages, and changes in clinical status and medication. Clinical and medication changes were always reported to the study’s clinician for confirmation and final recording in the online study’s management system.

At the end of the study, all examinations related to primary and secondary outcomes were repeated.

## Anticipated Results

**FIGURE 2:**
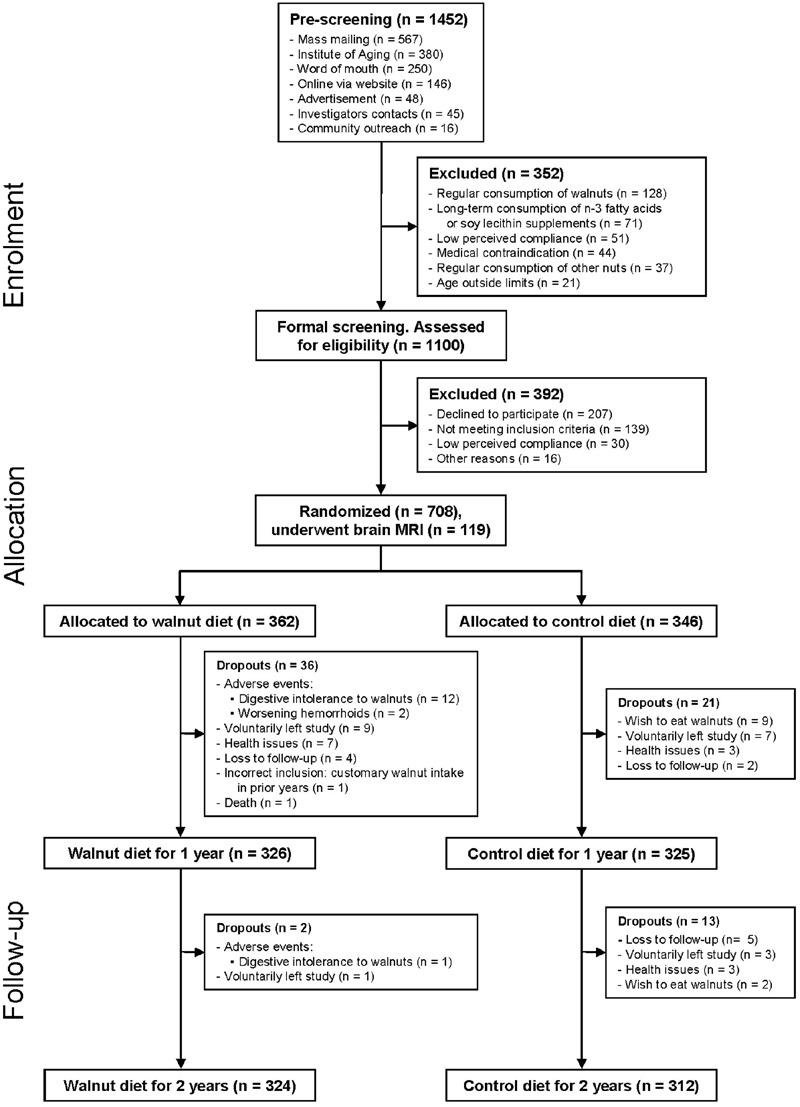
**Recruitment and participant allocation**.

Data collection for the WAHA study was completed May 31, 2016. As shown in **Figure 2**, of the total 708 randomized subjects, 92% (*n* = 651) completed the first year of the study, while 89.8% (*n* = 636) completed the 2 years. At the end of the study, the dropout rate is 10.2%. Attrition rates were similar in the two groups, *n* = 38 in the walnut group and *n* = 34 in the control group.

The baseline characteristics of the study participants by group are provided in **Table 2**. The mean age was 69.1 years and 67.9% were women. The characteristics of participants in the two intervention groups were well balanced except for smoking status, as there were more current smokers in the walnut group than in the control group.

**Table 2:** Baseline characteristics of 708 WAHA participants by treatment allocation.

Characteristics	Walnut	Control	*P*-value^∗^
No. (%)	362 (51.1)	346 (48.9)	
Enter with partner – no. (%)	98 (27.1)	88 (25.4)	0.62
Women – no. (%)	244 (67.4)	237 (68.5)	0.76
Age – year (mean ± SD)	69.4 ± 3.8	68.9 ± 3.5	0.07
Smoking – no. (%)			0.02
Never smoker	303 (83.7)	293 (84.7)	
Former smoker	43 (11.9)	49 (14.2)	
Current smoker	16 (4.4)	4 (1.2)	
Education – no. (%)			0.83
Basic (0–4 years)	11 (3.0)	8 (2.3)	
Elementary (5–8 years)	60 (16.6)	65 (18.8)	
Secondary (9–12 years)	70 (19.3)	65 (18.8)	
Post-secondary (>12 years)	221 (61.0)	208 (60.1)	
Height – cm	164.3 ± 9.5	163.3 ± 9.0	0.14
Weight – kg	73.8 ± 15.2	73.5 ± 14.7	0.78
Body mass index – kg/m^2^	27.2 ± 4.3	27.5 ± 4.4	0.42
Hypertension – no. (%)	191 (52.8)	183 (52.9)	0.97
Type-2 diabetes – no. (%)	37 (10.2)	33 (9.5)	0.76
Dyslipidemia – no. (%)	203 (56.1)	182 (52.6)	0.35

*Data are no. (%) or mean ± SD, as appropriate.*

*^∗^Obtained by Chi-square test or ANOVA, as appropriate.*

**FIGURE 3:**
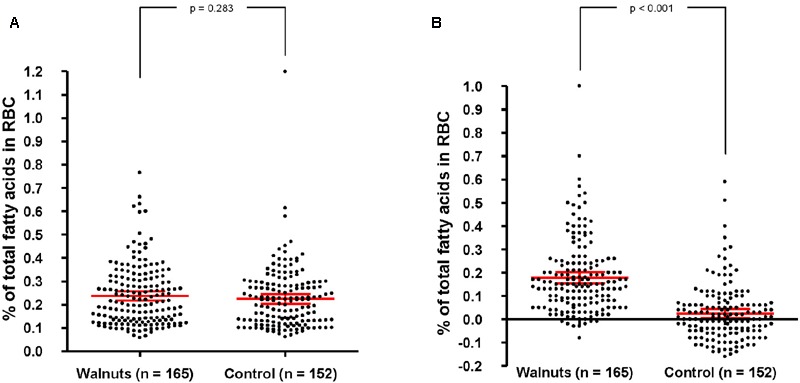
**Baseline biomarkers of adherence to walnuts and change after 1 year by intervention group.** RBC, red blood cells. In red, mean and 95% CI (ANCOVA, adjusting for center, age, and sex). **(A)** Percentage of total fatty acids in RBC at baseline. Values are 0.233 (0.218–0.247) and 0.221 (0.206–0.236) for the walnut and control groups, respectively. **(B)** Percentage of change of total fatty acids in RBC after 1 year. Values are 0.163 (0.144–0.183) and 0.019 (0.001–0.039) for the walnut and control groups, respectively.

To objectively determine adherence to supplemental walnuts, we measured the RBC proportions of ALA, a fatty acid characteristic of walnuts, at baseline and after 1 year of intervention in a random sub-sample of participants. There were no significant differences in RBC ALA between intervention groups at baseline (**Figure 3A**). After adjusting for center, age, and sex, 1-year changes in RBC ALA were increases of 0.16% of total fatty acids (95% confidence interval, 0.144–0.183) and 0.02% (95% confidence interval, 0.001–0.039) for the walnuts and control group, respectively (*P* between groups < 0.001) (**Figure 3B**).

## Potential Pitfalls and Counteracting Measures

The WAHA study was designed to assess whether a nutritional intervention with walnuts for 2 years can prevent cognitive impairment and preserve retinal health in a healthy elderly population.

An unavoidable limitation of the study is not being able to blind participants to the intervention since it consists of a whole food. Another limitation is the difficulty in keeping older, free-living volunteers in the study during 2 years and ensuring compliance with the high-fat supplemental food in the walnut diet group. The bimonthly visits by dietitians and their availability to answer questions related to diet or body weight and solve any problems with walnut ingestion contributed to ensure participants’ loyalty. Thus, the final dropout rate was only 10% and there was good compliance with both eating the walnuts in the walnut group and not eating them in the control group, as attested by 1-year changes of RBC ALA, an objective biomarker of walnut consumption. Finally, by the design of the recruitment methodology, self-selected participants are in better health and have a higher educational level than the general population, which could lead to lower than expected age-related cognitive decline and a lower probability of improving cognitive function with the intervention. The mean baseline age of nearly 70 years, however, may partly overcome this healthy participant effect.

The WAHA study has several strengths. This is the first RCT that examines the effects of a single whole food, walnuts, on cognitive performance and retinal health. By being an RCT the results of the main outcomes should provide a high level of evidence, while those of secondary outcomes, such as brain MRI, carotid ultrasonography, 24-h ambulatory blood pressure, inflammatory markers, and telomere length, might shed light on the mechanisms of a putative beneficial effect of the walnut diet on age-related disorders. Having participants from two clinical centers located in different geographical regions with variations in background diet and lifestyle habits increases the external validity. We assessed the primary outcome cognitive decline with a battery of standardized neuropsychological tests designed to evaluate different cognitive domains, making it a comprehensive approach. Changes in the retina were also evaluated by a precise quantitative technique such as OCT. Finally the free-living aspect of the study makes it as close to real-life as possible since there were no major diet changes to be made except to either eat walnuts daily or abstain from eating walnuts. The results should thus be applicable to a wider population and might have a significant impact on global public health recommendations.

In summary, the WAHA study evaluates cognitive function and retinal health in an elderly cohort following daily ingestion of walnuts for 2 years. The results might provide high-level evidence of the benefit of regular walnut consumption on delaying the onset of age-related degenerative diseases. The findings should translate into public health policy and sound recommendations to the general population.

## Ethics Statement

The study protocol was conducted in accordance with the guidelines of the Declaration of Helsinki and was approved by the institutional review boards of each center. All participants provided written informed consent.

## Author Contributions

ER and JS obtained funding and supervised the study. They also developed and drafted this study protocol in consultation with SR, CV-P, MC, MS-M, AP-H, RC-M, AS-V, and MD. SJ and MC were responsible for the recruitment process. CV-P and AA were in charge of cognitive assessment. Biochemical samples were managed and analyzed by SJ, MC, AS-V, and CC. MS-M, AP-H, IR, TF-S, EH, EB, NK, and LH were involved in dietary intervention and nutritional and anthropometric data collection. RC-M, SA, and JF were in charge of ophthalmologic assessment. MD and AL-I were responsible for clinical data, medical records, and blood pressure. All authors revised the manuscript critically for intellectual content and gave final approval of the version to be published.

## Conflict of Interest Statement

The Principal Investigators of the two centers (JS and ER) have received grants for research through their institutions from the California Walnut Commission and are non-paid members of its Scientific Advisory Committee. All the other authors declare that the research was conducted in the absence of any commercial or financial relationships that could be construed as a potential conflict of interest.

## Footnotes

1 https://clinicaltrials.gov/show/NCT01634841
